# Efficacies of Cabotegravir and Bictegravir against drug-resistant HIV-1 integrase mutants

**DOI:** 10.1186/s12977-018-0420-7

**Published:** 2018-05-16

**Authors:** Steven J. Smith, Xue Zhi Zhao, Terrence R. Burke, Stephen H. Hughes

**Affiliations:** 1 HIV Dynamics and Replication Program, National Cancer Institute-Frederick, National Institutes of Health, Frederick, MD USA; 2 Chemical Biology Laboratory, National Cancer Institute-Frederick, National Institutes of Health, Frederick, MD USA

**Keywords:** HIV-1, Integrase, Infectivity, Potency, Susceptibility, Modeling, Resistance

## Abstract

**Background:**

Integrase strand transfer inhibitors (INSTIs) are the class of antiretroviral (ARV) drugs most recently approved by the FDA for the treatment of HIV-1 infections. INSTIs block the strand transfer reaction catalyzed by HIV-1 integrase (IN) and have been shown to potently inhibit infection by wild-type HIV-1. Of the three current FDA-approved INSTIs, Dolutegravir (DTG), has been the most effective, in part because treatment does not readily select for resistant mutants. However, recent studies showed that when INSTI-experienced patients are put on a DTG-salvage therapy, they have reduced response rates. Two new INSTIs, Cabotegravir (CAB) and Bictegravir (BIC), are currently in late-stage clinical trials.

**Results:**

Both CAB and BIC had much broader antiviral profiles than RAL and EVG against the INSTI-resistant single, double, and triple HIV-1 mutants used in this study. BIC was more effective than DTG against several INSTI-resistant mutants. Overall, in terms of their ability to inhibit a broad range of INSTI-resistant IN mutants, BIC was superior to DTG, and DTG was superior to CAB. Modeling the binding of CAB, BIC, and DTG within the active site of IN suggested that the “left side” of the INSTI pharmacophore (the side away from the viral DNA) was important in determining the ability of the compound to inhibit the IN mutants we tested.

**Conclusions:**

Of the two INSTIs in late stage clinical trials, BIC appears to be better able to inhibit the replication of a broad range of IN mutants. BIC retained potency against several of the INSTI-resistant mutants that caused a decrease in susceptibility to DTG.

**Electronic supplementary material:**

The online version of this article (10.1186/s12977-018-0420-7) contains supplementary material, which is available to authorized users.

## Background

**Fig. 1:**
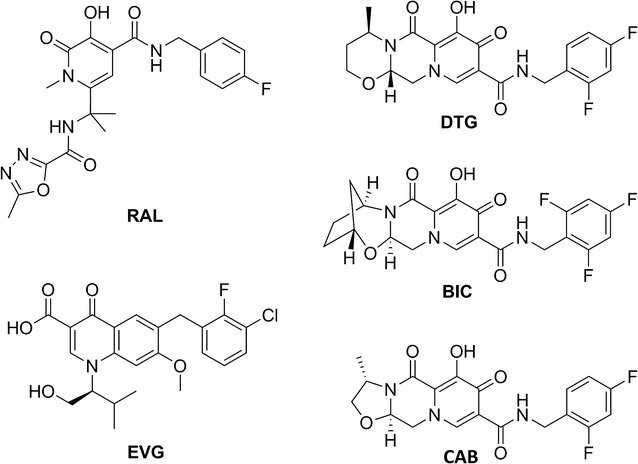
Chemical structures of INSTIs. The chemical structures of RAL, EVG, DTG, BIC, and CAB are shown

INSTIs are the class of antiretroviral (ARV) drugs most recently approved by the FDA to treat HIV-1 infections. INSTIs target the second reaction performed by HIV-1 Integrase (IN), strand transfer (ST), in which IN catalyzes the integration of the viral DNA into the cellular genome [1, 2]. INSTIs have a centralized pharmacophore, which contains a chelating functionality that interacts with the two catalytic Mg^2+^ ions at the IN active site [3, 4]. This central pharmacophore is joined to a halogenated benzyl moiety that interacts with the penultimate base at the 3′ end of the viral DNA [5]. Thus, INSTIs interact with both the enzyme and its nucleic acid substrate. The combination of these interactions allows the INSTIs to target and potently inhibit HIV-1 IN. Raltegravir (RAL) and Elvitegravir (EVG) are the first and second FDA-approved INSTIs, respectively. They potently inhibit WT HIV-1; however, resistant mutants can develop relatively quickly (Fig. 1). A partial list of the well-defined primary resistance mutations includes: Y143R, N155H, G140S/Q148H, T66I, and E92Q. Other mutations that confer resistance to RAL and EVG have been identified. In many cases, mutations selected by either RAL or EVG reduce the susceptibility of IN to the other INSTI, showing that RAL and EVG have overlapping resistance profiles [6–8].

In 2013, Dolutegravir (DTG) was approved by the FDA and it quickly became a preferred drug for combination antiretroviral therapy (cART) [9–12]. DTG differs from the first generation INSTIs in that its chelating motif is located on a tri-cyclic scaffold [13, 14]. In addition, the structural component that connects the central chelating moiety to the halogenated benzyl group is longer than it is in either RAL or EVG (Fig. 1) [15]. Not only do these structural differences allow DTG to be highly effective against WT HIV-1, but DTG is much more potent against IN mutants that confer resistance to the first generation INSTIs. Moreover, it has been difficult to select for DTG resistant mutants in cell culture and the treatment of HIV-1 patients using DTG has been, generally speaking, quite successful [16–21].

The usefulness of most ARV drugs is limited by the emergence of resistant mutants, and DTG will not be an exception. Recent in vitro selection studies with DTG have uncovered resistance mutations [22–24]. In clinical trials with INSTI-experienced subjects [25, 26] whose viruses had INSTI resistance mutations at the primary position Q148 plus at least one additional mutation at any of the secondary positions, L74, E138, G140, or G163, patients were put on a salvage regimen that included DTG. This change in therapy failed to lower HIV-1 below 50 copies/mL. Analysis of the virus present in the patients after the trial showed that additional mutations were selected in IN. These results showed that mutations that confer resistance to DTG can be selected in viruses that carry preexisting resistance mutations.

Recently, two new INSTIs, Cabotegravir (CAB) and Bictegravir (BIC), have been developed and these are currently in late phase clinical trials [13, 27, 28]. BIC and CAB, which are structurally similar to DTG, (both have tri-cyclic central pharmacophores), could offer therapeutic alternatives to HIV-1 patients (Fig. 1). Here, we describe evaluation of the antiviral potency of CAB and BIC against broad panels of well-characterized INSTI-resistant single and double mutants, and against the INSTI-resistant triple mutants identified in the VIKING clinical trials. Our objective was to determine how well these new INSTIs performed compared to DTG, the current standard of care.

## Results

### Initial screening of CAB and BIC against primary INSTI resistant mutants

**Fig. 2:**
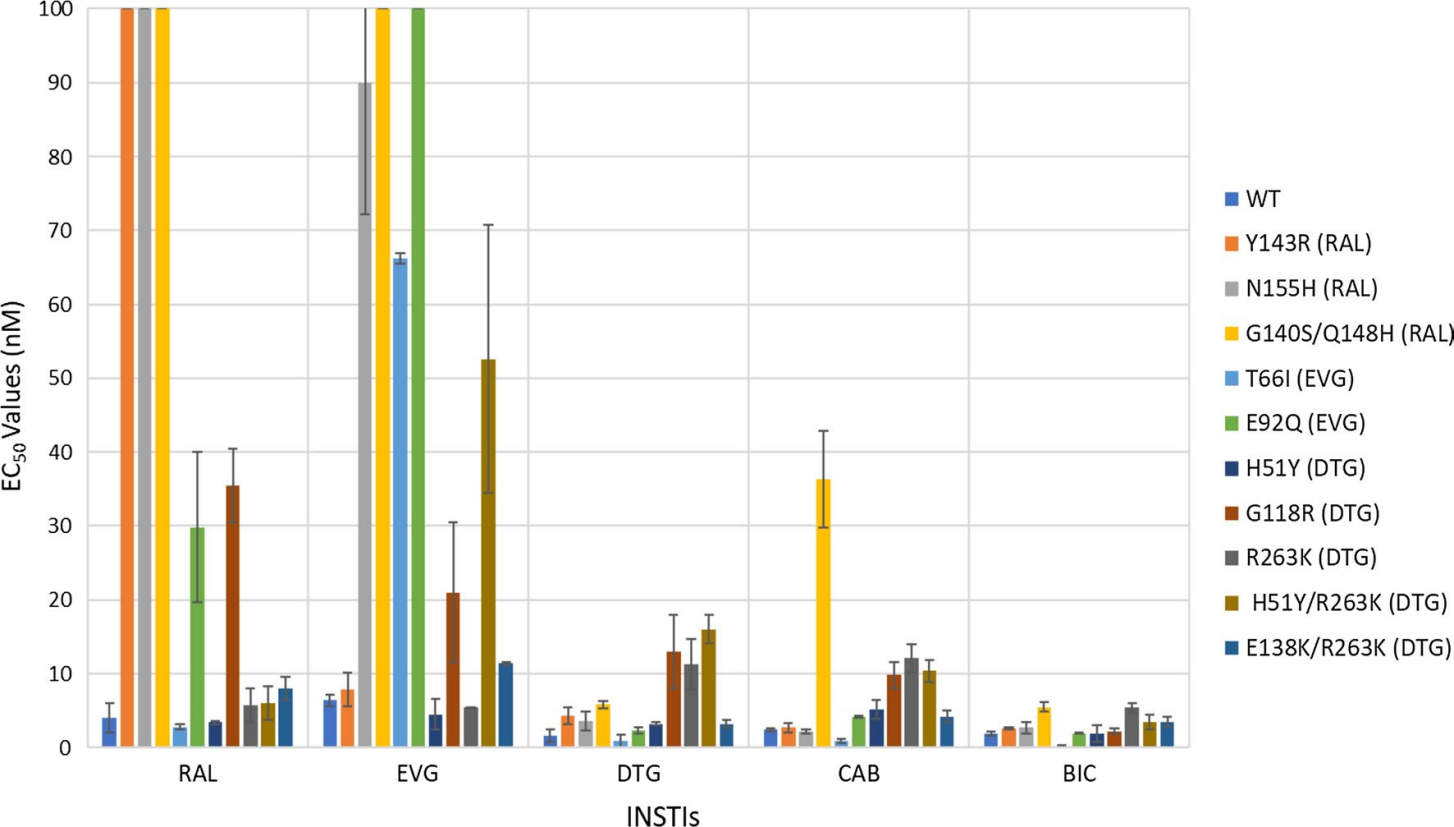
Antiviral activities of BIC and CAB against primary INSTI-resistant mutants. The EC_50_ values were determined, in single round infection assays, using vectors that carry the INSTI-resistant IN mutants. Error bars represent the standard deviations in the data from independent experiments (n = 4). The EC_50_ values shown in the figure have a maximum of 100 nM. The EC_50_ values of RAL against Y143R, N155H, and G140S/Q148H, EVG versus G140S/Q148H, and E92Q primary INSTI-resistant mutants were all > 100 nM

**Fig. 3:**
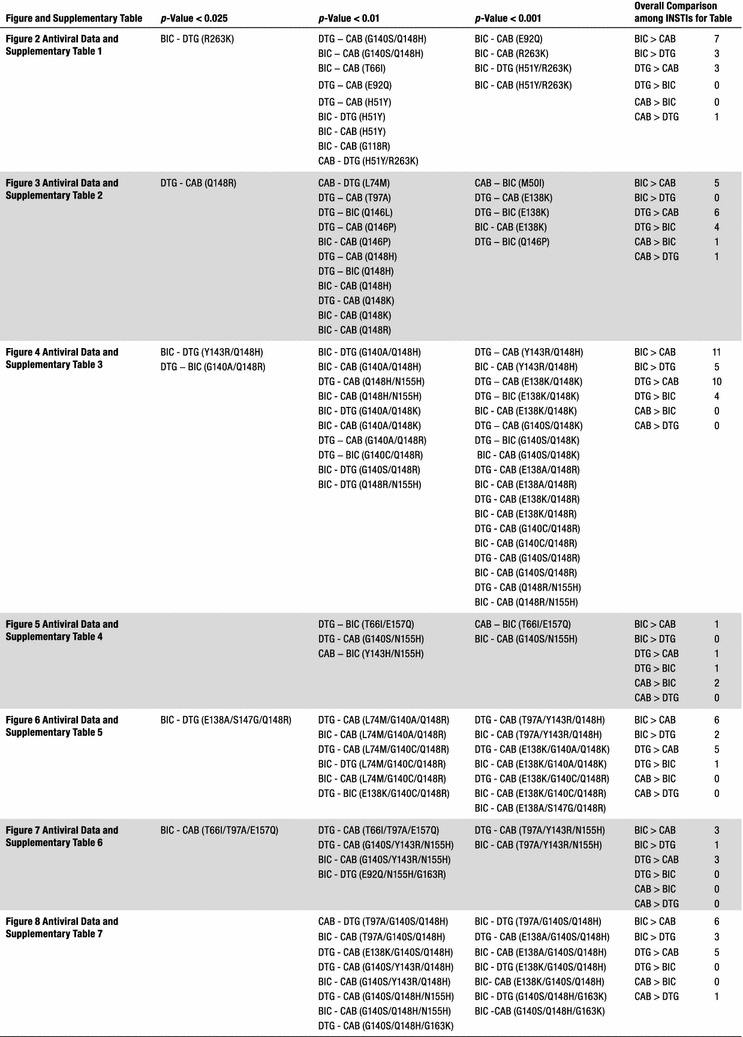
Statistical significance of the antiviral data among DTG, CAB, and BIC. The Student’s *t* test was used to calculate the statistical significance of the differences in the antiviral activities of the INSTIs. Because of multiple comparisons, *p* values < 0.025 were considered statistically significant when comparing the efficacies among DTG, CAB, and BIC

The abilities of CAB and BIC to inhibit the replication of WT HIV-1 and INSTI-resistant mutants were determined in single-round viral replication assays. We initially screened CAB and BIC against a panel of primary INSTI-resistant mutants, which included: Y143R, N155H, G140S/Q148H, T66I, E92Q, H51Y, G118R, R263K, H51Y/R263K, and E138K/E263K. Y143R, N155H, and G140S/Q148H (Fig. 2; see also Additional file 1: Table S1A) were chosen because they have been selected in patients by treatment with RAL [29–31]; the T66I and E92Q mutants were selected by treatment with EVG [32–34]. The IN mutations H51Y, G118R, R263K, H51Y/R263K, E138K/R263K mutants have been selected with DTG in cell culture [22–24]. The R263K mutation has been selected in several treatment-experienced, INSTI-naïve patients undergoing DTG therapy [16]. Both CAB and BIC potently inhibited the infection of WT HIV-1 with EC_50_ values equivalent to the FDA-approved INSTIs (< 3 nM). Moreover, CAB and BIC were minimally toxic in cell culture assays with CC_50_ values > 250 µM (data not shown), which is similar to the FDA-approved INSTIs. This demonstrates that these INSTIs have very favorable therapeutic indexes in cultured cells. Additionally, both CAB and BIC potently inhibited the RAL-resistant mutants Y143R and N155H; the EVG-resistant IN mutants T66I and E92Q, and the DTG-resistant IN mutant H51Y and E138K/R263K with EC_50_ values < 5 nM. However, only BIC potently inhibited the well-known RAL-resistant IN double mutant G140S/Q148H and the DTG-resistant IN mutants G118R, R263K, and H51Y/R263K with EC_50_ values ≤ 5 nM. The RAL-resistant IN mutant G140S/Q148H caused a substantial loss of CAB potency (36.3 ± 6.5 nM), while there was a smaller but still modest loss of potency against the DTG-resistant IN mutants G118R (12.1 ± 1.9 nM), R263K (13.4 ± 1.3 nM), and H51Y/R263K (10.4 ± 1.5 nM). These antiviral data were compared to previous screens, in which the antiviral potencies of RAL, EVG, and DTG were measured against the same INSTI-resistant primary mutants [35, 36]. When the antiviral profiles of the second generation INSTIs, DTG, CAB and BIC were compared to the FDA-approved INSTIs for WT HIV-1 and the RAL- and EVG-resistant mutants, all of the second generation INSTIs had antiviral profiles that were obviously superior to RAL and EVG. The differences were sufficiently clear cut that the comparisons between the first and second generation INSTIs were not subjected to statistical analysis. The more important question was whether either CAB or BIC was better than DTG, in terms of their ability to inhibit the IN mutants. To make the comparison objective, the statistical significance of the EC_50_ data for CAB, BIC, and DTG were analyzed using the Student’s *t* test. The EC_50_ values for WT HIV for DTG, CAB and BIC were similar, which allowed us to compare the EC_50_ values for the mutants directly. In the initial screen, which included ten INSTI-resistant primary mutants, BIC was significantly better than CAB for seven of these ten primary mutants (four *p* values < 0.01 and three *p* values < 0.001; see Fig. 3 and Additional file 1: Table S1B). In addition, BIC was better than DTG against three of the primary mutants. In contrast, CAB was significantly better than DTG for one primary mutant and DTG was better than CAB for three of the primary mutants.

### Antiviral activities of CAB and BIC against other common INSTI-resistant single mutants

**Fig. 4:**
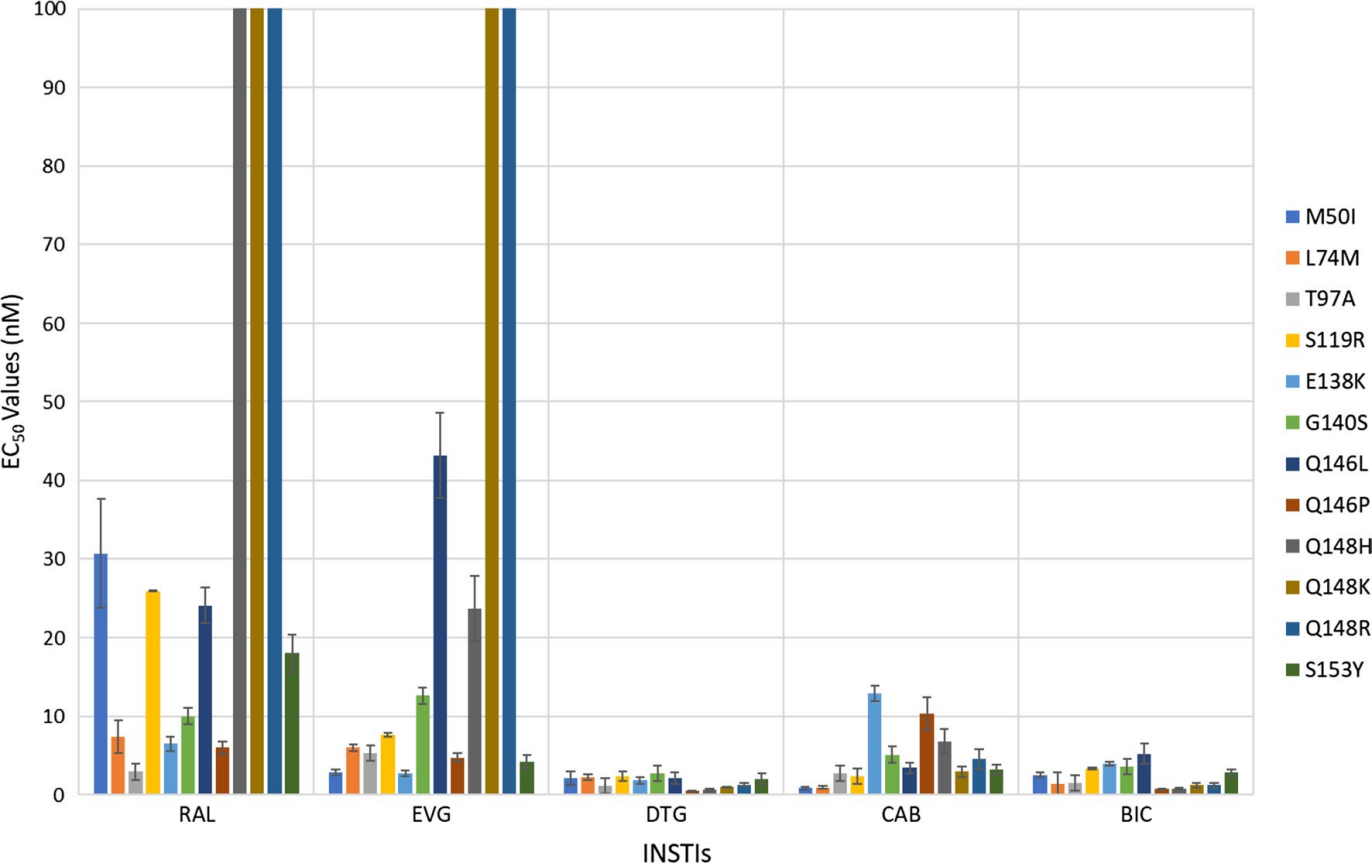
Antiviral activities of BIC and CAB against common INSTI-resistant single mutants. The EC_50_ values were determined using vectors that carry the INSTI-resistant IN double mutants in single round infection assays. Error bars represent the standard deviations in the data from independent experiments (n = 4). The EC_50_ values shown in the figure have a maximum of 100 nM. The EC_50_ values of RAL against Q148H, Q148K, and Q148R and EVG versus Q148K and Q148R INSTI-resistant mutants were all > 100 nM

We determined the antiviral profiles of CAB and BIC, as well as the FDA-approved INSTIs, against a second panel of additional INSTI-resistant single mutants to compare the strengths and weaknesses of the two new INSTIs and the FDA-approved INSTIs [37–39]. This panel of INSTI-resistant single mutants included: M50I, L74M, T97A, S119R, E138K, G140S, Q146L, Q146P, Q148H, Q148K, Q148R, and S153Y (Fig. 4; Additional file 1: Table S2A). BIC potently inhibited this entire panel of INSTI-resistant mutants with EC_50_ values below 5 nM, which was comparable to DTG. CAB also inhibited the majority of mutants in this panel. However, it lost some potency against the INSTI-resistant single mutants E138K (12.9 ± 1.0 nM), Q146P (10.3 ± 2.1 nM), and Q148H (6.8 ± 1.5 nM). Most of the INSTI-resistant single mutants in this panel caused significant drops in susceptibility to the first generation INSTIs, RAL and EVG, with the Q148H/K/R mutants having the greatest effect on the EC_50_ values. Based on the data obtained with the mutants in this panel, DTG was better than CAB and BIC (Fig. 3; Additional file 1: Table S2B). DTG was significantly better than CAB against six of the mutants and better than BIC against four mutants (two *p* values < 0.001). Conversely, BIC was better than CAB against five of these mutants.

#### Antiviral activities of CAB and BIC against a panel of INSTI-resistant double mutants having a primary mutation at position Q148

**Fig. 5:**
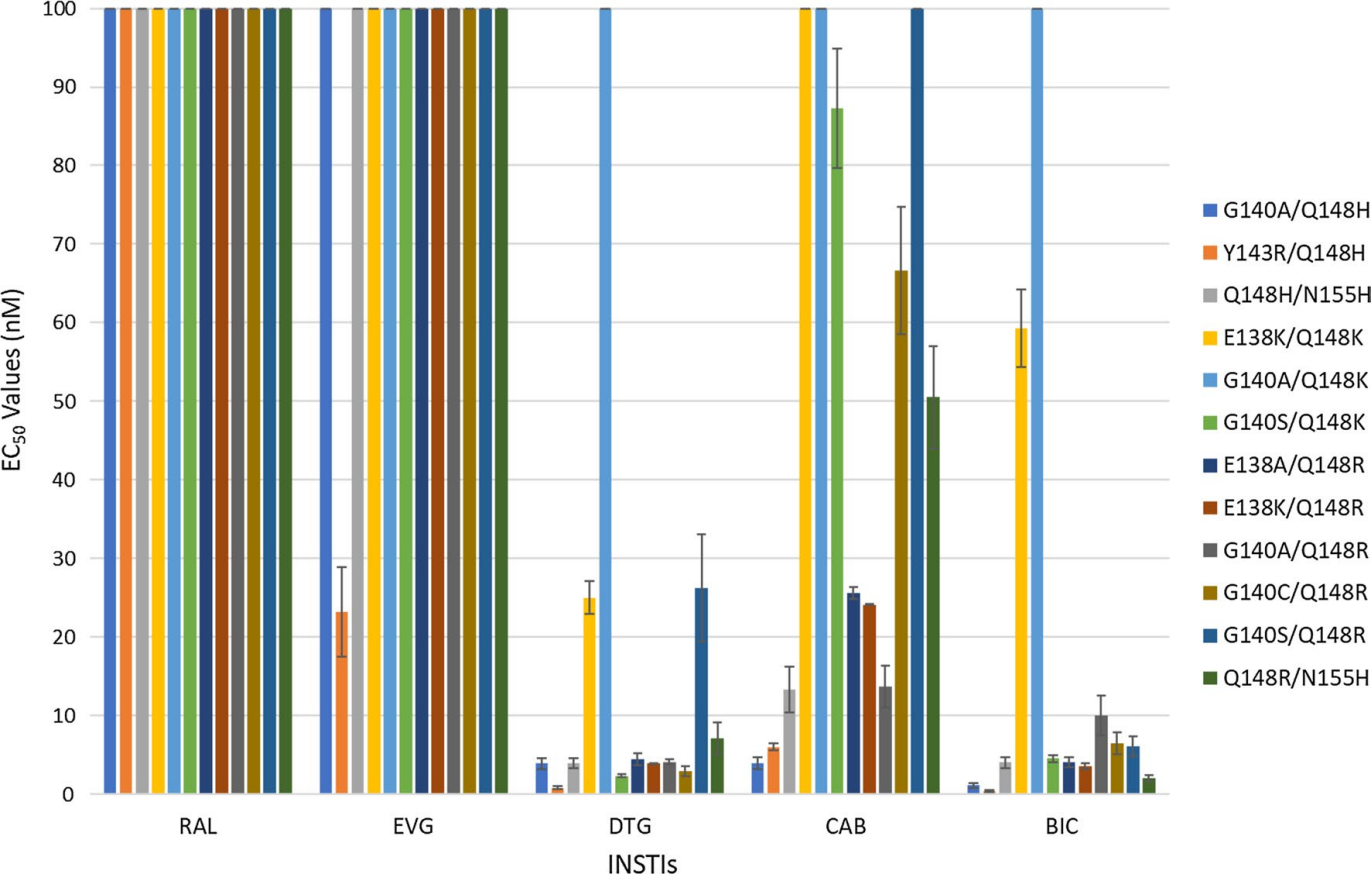
Antiviral activities of BIC and CAB against a panel of INSTI-resistant double mutants that have a primary mutation at position Q148. The EC_50_ values were determined using vectors that carry the INSTI-resistant double mutants in single round infection assays. Error bars represent the standard deviations in the data from independent experiments (n = 4). The EC_50_ values shown in the figure have a maximum of 100 nM. The EC_50_ values of RAL and EVG against this entire panel (except for EVG versus Y143R/Q148H) were all > 100 nM. The EC_50_ values of DTG against G140A/Q148K, CAB versus E138K/Q148K, G140A/Q148K, G140S/Q148R, and BIC against G140A/Q148K were all > 100 nM

We next tested CAB, BIC, and the FDA-approved INSTIs against a panel of INSTI-resistant double mutants that included either one of the primary mutations at position Q148 (H/K/R), or Y143R or N155H. These primary mutations were combined with a secondary mutation at positions E138 (A/K) or G140 (A/C/S) (Fig. 5; Additional file 1: Table S3A). BIC potently inhibited (EC_50_ < 5 nM) the INSTI-resistant double mutants G140A/Q148H, Y143R/Q148H, Q148H/N155H, G140S/Q148K, E138A/Q148R, E138K/Q148R, and Q148R/N155H. Conversely, the INSTI-resistant double mutants G140A/Q148R (10 ± 2.5 nM), G140C/Q148R (6.4 ± 1.4 nM), G140S/Q148R (6.1 ± 1.3 nM) caused small losses in susceptibility to BIC, whereas the double mutants E138K/Q148K (59.3 ± 4.9 nM) and G140A/Q148K (137.1 ± 5.0 nM) resulted in substantial reductions in susceptibility to BIC. However, there was a large reduction in CAB potency against most of the double mutants in the panel. The double mutants Y143R/Q148H (6.0 ± 0.4 nM), E138A/Q148R (25.6 ± 0.8 nM), E138K/Q148R (24.1 ± 0.1 nM), and G140A/Q148R (13.7 ± 2.7 nM) caused a minimal loss in susceptibility to CAB, whereas E138K/Q148K (772.1 ± 72.2 nM), G140A/Q148K (393.1 ± 51.1 nM), G140S/Q148K (87.3 ± 7.6 nM), G140C/Q148R (66.6 ± 8.1 nM), and Q148R/N155H (50.5 ± 6.5 nM) caused large reductions in susceptibility to CAB. DTG was very effective across this panel of INSTI-resistant double mutants. However, it sustained a moderate loss in potency against the INSTI-resistant double mutant E138K/Q148K (25.0 ± 2.1 nM) and significant loss in potency against the INSTI-resistant double mutant G140A/Q148K (450.7 ± 58.8 nM). The first generation INSTIs RAL and EVG exhibited considerable loss of potency against all of the mutants in this panel of INSTI-resistant double mutants. BIC was significantly better than DTG against five of these double mutants (four *p* values < 0.01; Fig. 3; Additional file 1: Table S3B); however, DTG was better than BIC for four of the mutants (two *p* values < 0.001). CAB was not significantly better than either DTG or BIC against any double mutants.

#### Antiviral activities of CAB and BIC against a panel of INSTI-resistant double mutants that included the primary mutations T66I and N155H and additional mutations at other positions

**Fig. 6:**
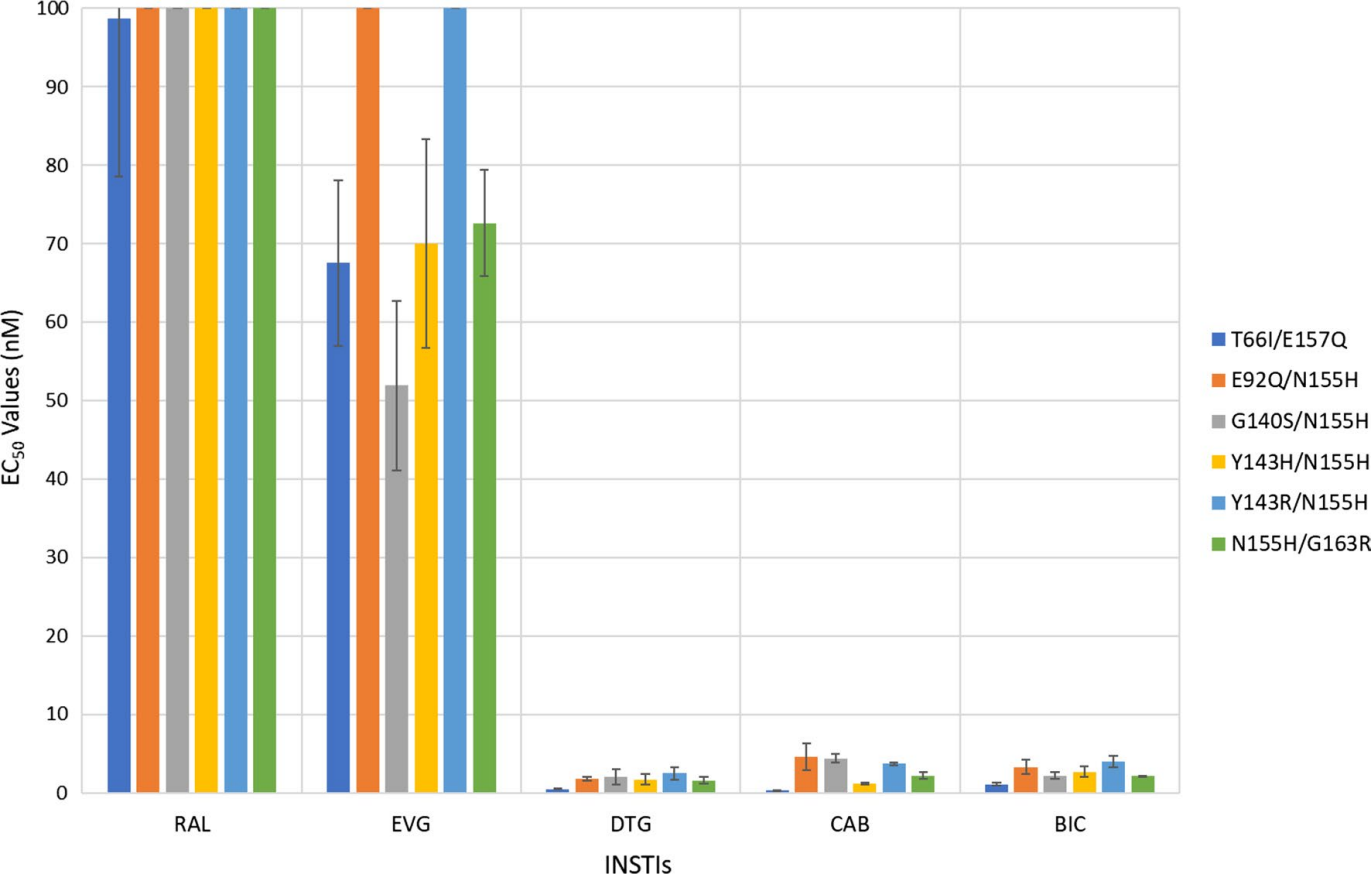
Antiviral activities of BIC and CAB against a panel of INSTI-resistant double mutants that included the primary mutations T66I and N155H with additional mutations at other positions. The EC_50_ values were determined using vectors that carry the INSTI-resistant double mutants in single round infection assays. Error bars represent the standard deviations in the data from independent experiments (n = 4). The EC_50_ values shown in the figure have a maximum of 100 nM. The EC_50_ values of RAL against this entire panel of INSTI-resistant double mutants were all > 100 nM. The EC_50_ values of EVG against E92Q/N155H and Y143R/N155H were all > 100 nM

**Fig. 7:**
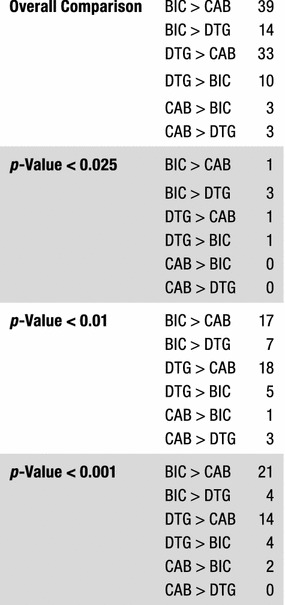
Overall Comparison of the statistical significance of the antiviral data among DTG, CAB, and BIC. The Student’s *t* test was used to calculate the statistical significance of the differences in the antiviral activities of the INSTIs. The *p* values < 0.025, < 0.01, and < 0.001 between DTG, CAB, and BIC were used to decide which INSTIs were more broadly efficacious against the mutants

We determined the antiviral profiles of CAB, BIC, and the FDA-approved INSTIs against the EVG-resistant double mutant T66I/E157Q and a panel of INSTI-resistant double mutants with a primary mutation at N155H and one of the following secondary mutations: E92Q, G140S, Y143H/R, or G163R (Fig. 6; Additional file 1: Table S4A). BIC, CAB, and DTG retained potency against the INSTI-resistant double mutant T66I/E157Q and the other INSTI-resistant double mutants (EC_50_ < 5 nM). The first generation INSTIs, RAL and EVG, failed to potently inhibit any of these double mutants. Based on this panel of mutants, the antiviral profiles of the three second generation INSTIs were similar to each other. CAB was significantly better than BIC against two of these double mutants (one *p* value < 0.001; see Figs. 3 and 7 and Additional file 1: Table S4B). DTG had better activity against one of the double mutants than CAB and BIC (*p* value < 0.01).

#### Antiviral activities of CAB and BIC against a panel INSTI-resistant triple mutants that included a primary mutation (Q148H/K/R) and two additional mutations

**Fig. 8:**
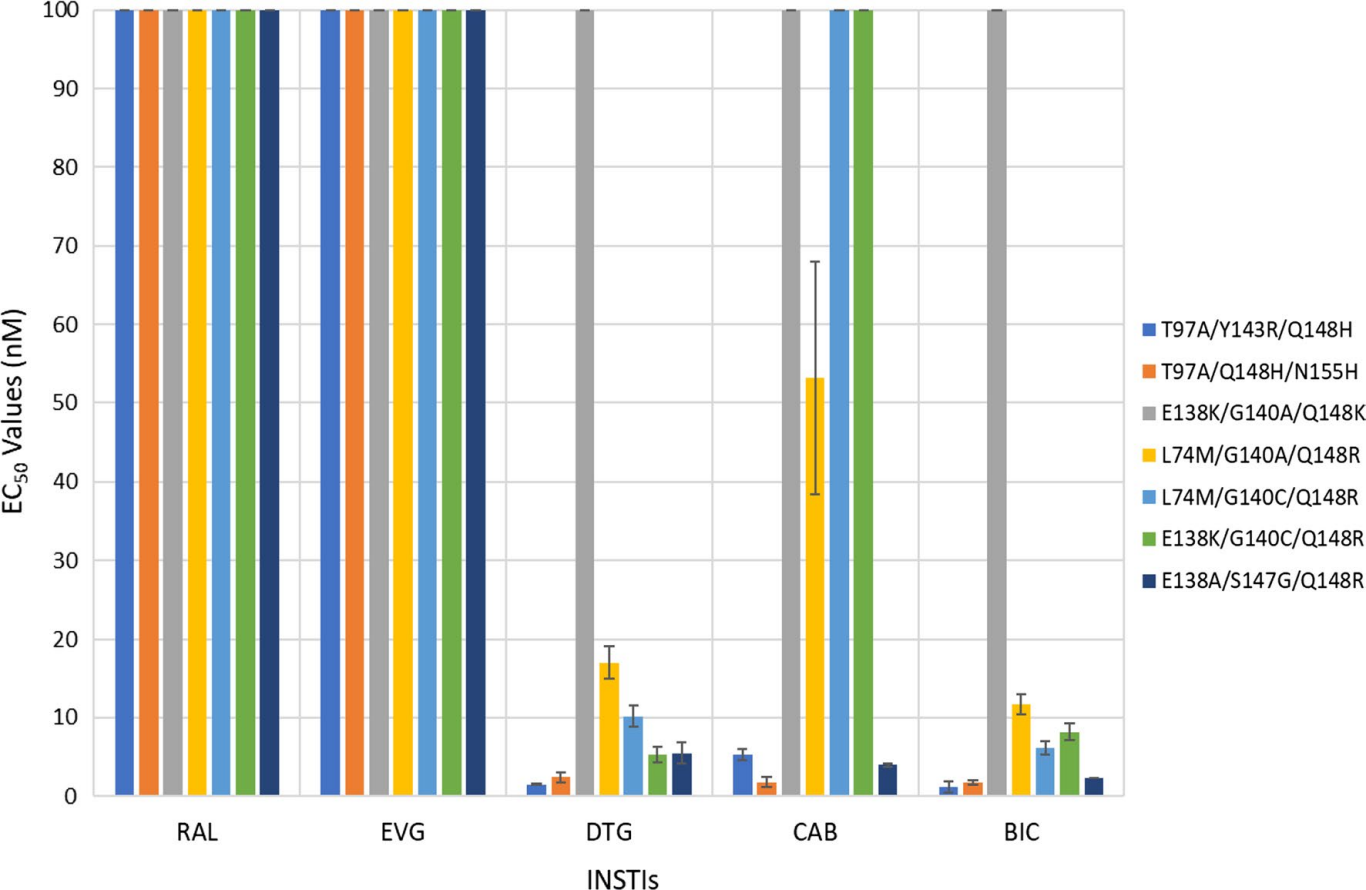
Antiviral activities of BIC and CAB against a panel INSTI-resistant triple mutants that included a primary mutation (Q148H/K/R) and two additional mutations. The EC_50_ values were determined using vectors that carry the INSTI-resistant triple mutants in single round infection assays. Error bars represent the standard deviations in the data from independent experiments (n = 4). The EC_50_ values shown in the figure have a maximum of 100 nM. The EC_50_ values of RAL and EVG versus this entire panel of INSTI-resistant triple mutants were all > 100 nM. The EC_50_ values of DTG against E138K/G140A/Q148K, CAB versus E138K/G140A/Q148K, L74M/G140C/Q148R, and E138K/G140C/Q148R, and BIC against E138K/G140A/Q148K were all > 100 nM

We determined the antiviral activities of CAB, BIC, and the FDA-approved INSTIs against a panel of INSTI-resistant triple mutants that included a primary mutation at Q148 (H/K/R) with two additional mutations at primary or secondary positions. The panel of INSTI-resistant triple mutants included: T97A/Y143R/Q148H, T97A/Q148H/N155H, E138K/G140A/Q148K, L74M/G140A/Q148R, L74M/G140C/Q148R, E138K/G140C/Q148R, and E138A/S147G/Q148R (Fig. 8; Additional file 1: Table S5A). Overall, DTG and BIC showed similar antiviral profiles against these triple mutants, and in some cases, CAB also retained potency. DTG, BIC, and CAB potently inhibited (EC_50_s ≤ 5 nM) the T97A/Y143R/Q148H and E138A/S147G/Q148R INSTI-resistant triple mutants. The E138K/G140C/Q148R INSTI-resistant mutant caused only a small loss of potency to DTG (5.3 ± 1.0 nM) and BIC (8.2 ± 1.1 nM). This mutant showed a significant reduction in susceptibility to CAB (134.2 ± 0.3 nM). The L74M/G140C/Q148R triple mutant was moderately susceptible to BIC (6.1 ± 0.9 nM) and DTG (10.2 ± 1.3 nM). However, this mutant caused a massive loss in susceptibility to CAB (220.3 ± 41.2 nM). The L74M/G140A/Q148R triple mutant with a different mutation at position G140, caused a modest loss of susceptibility to both DTG (12.0 ± 2.1 nM) and BIC (11.7 ± 1.3 nM); however, this also caused a substantial loss in susceptibility to CAB (53.2 ± 14.8 nM). Finally, DTG, BIC, and CAB failed to retain potency against the E138K/G140A/Q148K INSTI-resistant triple mutant (EC_50_s > 200 nM). The first generation INSTIs, RAL and EVG failed to retain potency against the entire panel of INSTI-resistant triple mutants, except for T97A/Y143R/Q148H, against which EVG showed modest inhibition, with an EC_50_ value of 41.6 ± 3.0 nM. BIC had significantly higher potencies against two of the INSTI-triple mutants than DTG (one *p* value < 0.01, see Fig. 3 and Additional file 1: Table S5B), compared to one for DTG versus BIC against the triple mutants in this panel. Both BIC and DTG were more effective than CAB. BIC had better efficacies than CAB against six of the triple mutants (four *p* values < 0.001); DTG was better than CAB for five of the mutants in this panel (three *p* values < 0.001).

#### Antiviral activities of CAB and BIC versus a panel of INSTI-resistant triple mutants that consists of a primary mutation at T66I and N155H with additional secondary mutations

**Fig. 9:**
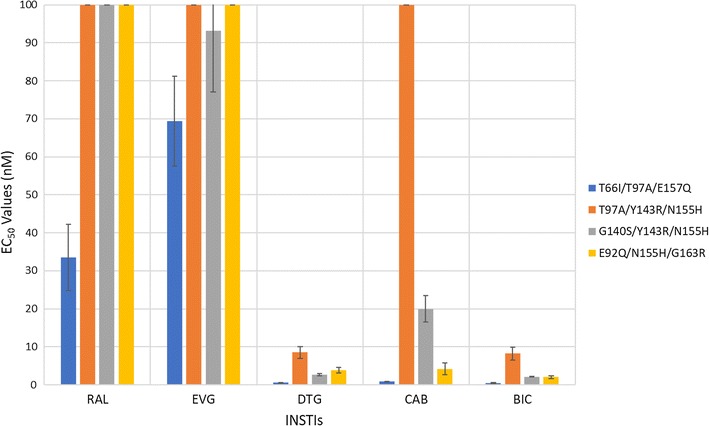
Antiviral activities of BIC and CAB versus a panel of INSTI-resistant triple mutants that consists of a primary mutation at T66I and N155H with additional secondary mutations. The EC_50_ values were determined using vectors that carry the INSTI-resistant triple mutants in single round infection assays. Error bars represent the standard deviations of the data from independent experiments (n = 4). The EC_50_ value shown in the figure have a maximum of 100 nM. The EC_50_ values of RAL against T97A/Y143R/N155H, G140S/Y143R/N155H, and E92Q/N155H/G163R were all > 100 nM. The EC_50_ values of EVG versus T97A/Y143R/N155H and E92Q/N155H/G163R and CAB against T97A/Y143R/N155H were all > 100 nM

We examined CAB, BIC, and the FDA-approved INSTIs, against a panel of INSTI-triple mutants that included T66I/T97A/E157Q, T97A/Y143R/N155H, G140S/Y143R/N155H, and E92Q/N155H/G163R (Fig. 9; Additional file 1: Table S6A). The triple mutant T66I/T97A/E157Q is an EVG-resistant mutant and, as expected, this mutant showed a substantial decrease in potency to EVG (69.4 ± 11.8 nM) and a lesser loss of potency to RAL (33.5 ± 8.7 nM). In contrast, DTG (0.5 ± 0.1 nM), BIC (0.4 ± 0.2 nM), and CAB (0.8 ± 0.1 nM) retained full potency against this triple mutant. Additionally, DTG, BIC, and CAB retained high antiviral potencies against the E92Q/N155H/G163R INSTI-resistant triple mutant (EC_50_ < 5 nM). The G140S/Y143R/N155H triple mutant was susceptible to both DTG (2.6 ± 0.3 nM) and BIC (2.1 ± 0.1 nM), but it caused a moderate loss in potency to CAB (20.0 ± 3.5 nM). Both DTG and BIC retained significant potency against the T97A/Y143R/N155H triple mutant, 8.5 ± 1.5 nM and 8.2 ± 1.7 nM, respectively, whereas CAB lost substantial potency (142.2 ± 8.3 nM). RAL and EVG both failed to potently inhibit the T97A/Y143R/N155H, G140S/Y143R/N155H, and E92Q/N155H/G163R INSTI-resistant triple mutants (EC_50_s > 90 nM). Both DTG and BIC were more effective than CAB; each one had a significantly higher potency than CAB against 3 of the triple mutants in this panel (at least one *p* value < 0.001; see Fig. 3 and Additional file 1: Table S6B). BIC was significantly better than DTG against one of the triple mutants (*p* value < 0.01).

#### Antiviral activities of CAB and BIC against a panel of INSTI-resistant triple mutants that include the well-characterized RAL-resistant double mutant G140S/Q148H and an additional secondary mutation

**Fig. 10:**
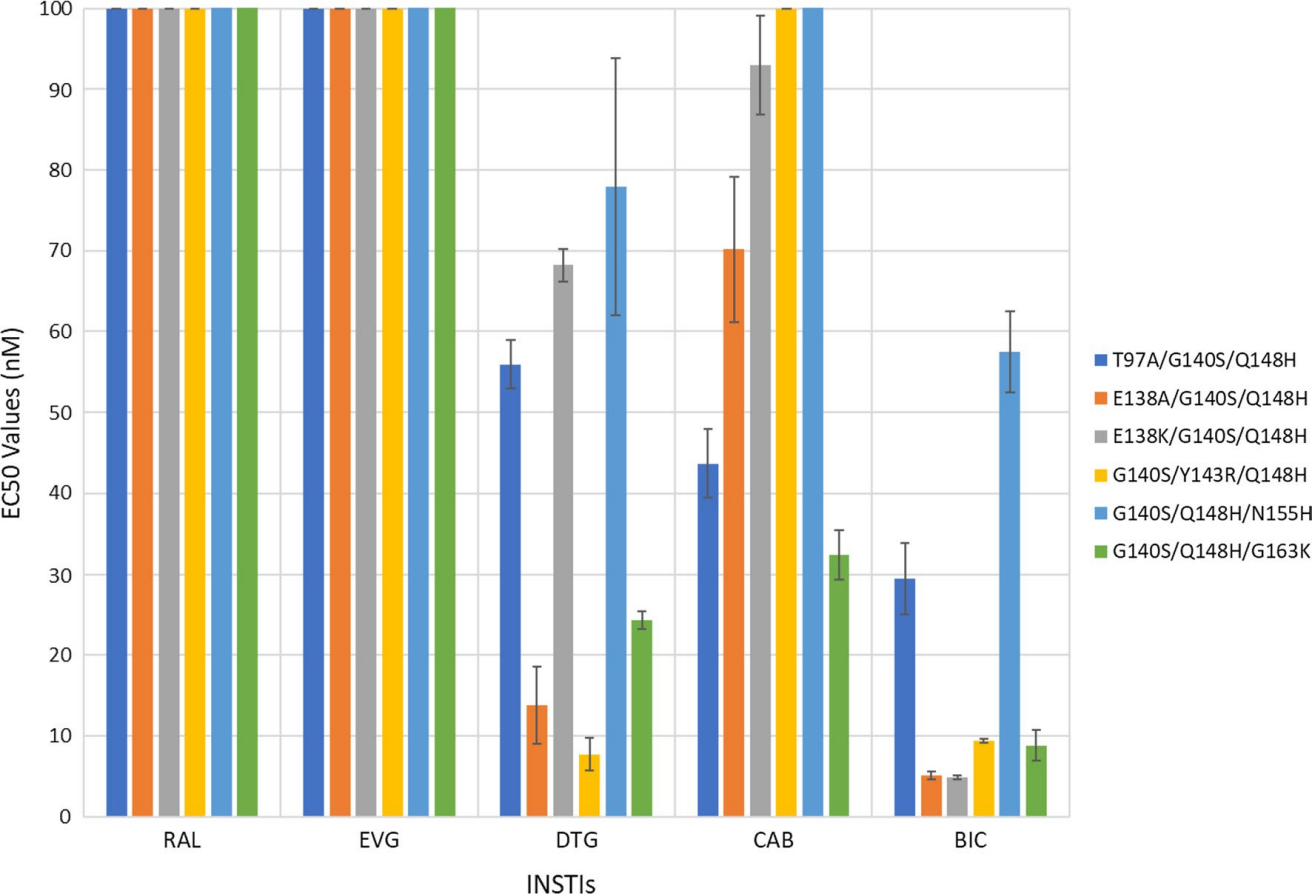
Antiviral activities of CAB and BIC against a panel of INSTI-resistant triple mutants that include the well-characterized RAL-resistant double mutant G140S/Q148H plus an additional secondary mutation. The EC_50_ values were determined using vectors that carry the INSTI-resistant triple mutants in single round infection assays. Error bars represent the standard deviations in the data from independent experiments (n = 4). The EC_50_ values shown in the figure have a maximum of 100 nM. The EC_50_ values of RAL and EVG against this entire panel of INSTI-resistant triple mutants were all > 100 nM. The EC_50_ values of CAB against G140S/Y143R/Q148H and G140S/Q148H/N155H were all > 100 nM

We tested the antiviral potencies of CAB, BIC, and the FDA-approved INSTIs against a panel of INSTI-resistant triple mutants that included the RAL-resistant G140S/Q148H double mutations with an additional mutation: T97A, E138A/K, Y143R, N155H, or G163K (Fig. 10; Additional file 1: Table S7A). As expected, both of the first generation INSTIs, RAL and EVG, were ineffective against this panel of six INSTI-resistant triple mutants (EC_50_s > 5000 nM). In addition, DTG, which potently inhibited the G140S/Q148H INSTI-resistant double mutant (EC_50_ < 5 nM) showed a loss of potency against the INSTI-resistant triple mutants. The E138A/G140S/Q148H, G140S/Y143R/Q148H, and G140S/Q148H/G163K triple mutants caused modest drops in potency, from 13.8 ± 4.8 nM, to 7.7 ± 2.0 nM, and 24.3 ± 1.1 nM, respectively. However, the INSTI-resistant triple mutants T97A/G140S/Q148H, E138K/G140S/Q148H, and G140S/Q148H/N155H caused substantial reductions in potency (EC_5_s ≥ 55 nM). CAB was not broadly active against these INSTI-resistant triple mutants; most of the mutants caused significant drops in susceptibility to CAB. Conversely, for this panel of mutants, BIC was more effective than DTG in retaining potency. BIC showed at most a modest loss in potency against E138A/G140S/Q148H, E138K/G140S/Q148H, G140S/Y143R/Q148H, and G140S/Q148H/G163K (EC_50_s < 10 nM. However, the INSTI-resistant triple mutant T97A/G140S/Q148H caused a larger reduction in susceptibility to BIC (29.5 ± 4.4 nM). Thus, BIC was superior to the other INSTIs in terms of its ability to retain antiviral activity against this set of triple mutants. BIC was the superior INSTI against this panel of triple mutants, as it had significantly better potencies against five INSTI-resistant triple mutants than CAB (2 *p* values < 0.01 and 3 *p* values < 0.001, see Fig. 3 and Additional file 1: S7B) and three triple mutants than DTG (*p* values < 0.001). DTG was a better INSTI than CAB as it had higher efficacies against five triple mutants than CAB.

#### Homology modeling of the binding of BIC and CAB into the HIV-1 IN active site using PFV intasome structural data

**Fig. 11:**
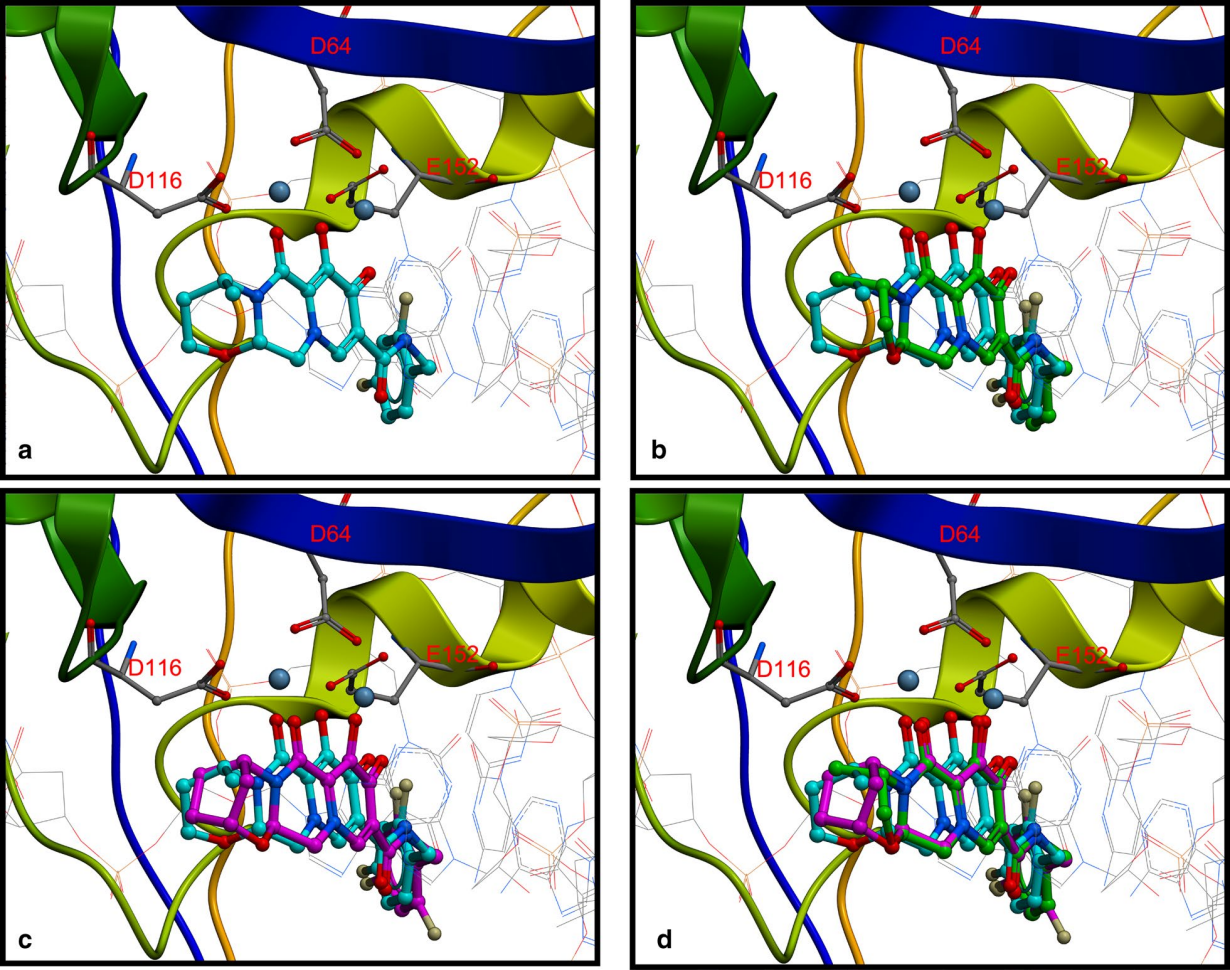
Modeling BIC and CAB into the PFV Intasome. The four panels show models of DTG, CAB, or BIC bound in the active site of HIV-1 IN. The upper left panel **a** shows a model of DTG (cyan) bound to HIV-1 IN. The upper right panel **b** shows a model of CAB (green) bound to the active site of the HIV-1 IN using DTG (cyan) as the template. The lower left panel **c** shows a model of BIC (magenta) bound to the active site of HIV-1 IN using DTG (cyan) as the template. The lower right panel **d** shows an overlay of the binding of DTG (cyan), BIC (magenta), and CAB (green) to HIV-1 IN, specifically showing how the “left-side” of these three INSTIs, the part of pharmacophores distal from the end of the viral DNA, interact with HIV-1 IN. All four panels show the Mg^2+^ cofactors rendered in space-filling format (slate gray) interacting with the chelating motifs of each of the INSTIs. HIV-1 IN is depicted in multi-colored ribbons with active site residues D64, D116, and E152 labeled in red and rendered in dark gray ball and stick format

Using the previously reported crystal structure of the PFV intasome with DTG bound at the catalytic site (PDB ID: 3S3M) [15] and the structure of the HIV-1 IN strand transfer complex (STC) solved by electron microscopy as a template (PDB ID: 5U1C) [40], homology models were prepared of BIC and CAB bound to the HIV-1 intasome (Fig. 11, panels B and C). Modeling allowed us (1) to understand better how BIC and CAB bind in the HIV-1 IN active site and (2) to identify structural features that may help (or hinder) these INSTIs in overcoming INSTI-resistant mutants. The chelating motifs of BIC and CAB aligned similarly to DTG (Fig. 11, panel D), as did the halobenzyl moieties, which have π–π hydrophobic stacking interactions with the penultimate cytosine on the 3′ ends of the viral DNA. However, it is the “left” side of the tricyclic ring system of BIC, which is the portion of the INSTI that is distal to the end of the viral DNA, and has an oxazepine ring which features a methylene bridge and lacks a methyl group, that appears to distinguish BIC from DTG. This cyclic modification can, in the model, adopt and maintain a different configuration from the components on the left side of DTG and CAB. Both the methyl-modified oxazine ring of DTG and the methyl-modified oxazole ring of CAB appear to be more constrained than the oxazepine ring of BIC. The greater flexibility of the oxazepane ring allows it to bend backwards or forwards, depending on the exact geometry of the active site, which can be modified by nearby mutations. Thus, the apparent greater conformational flexibility of the oxazepine ring could allow BIC to bind tightly to the various INSTI-resistant mutants, such as G118R and S119R, which affect the periphery of the IN active site, and limit the modifications that can be added to the “left” side of the INSTI scaffold distal to the end of the viral DNA (unpublished observations). Conversely, the oxazole ring of CAB is pointed out and away from the position occupied by the corresponding oxazine ring of DTG (Fig. 11, panel B), and its methyl group does not appear to be in a position to make an important contribution to binding, which is in good agreement with the data of Yoshinaga et al. [41], which appeared when this manuscript was in review. This could account for the fact that, although CAB and DTG adopt similar spatial orientations when bound to the IN active site, DTG is much more broadly effective against INSTI-resistant mutants (see “Discussion”).

## Discussion

The relatively recent development of INSTIs as potent and effective HIV-1 inhibitors permits improved treatment strategies for HIV-1 infected patients. In general, INSTIs are minimally toxic and work well in combination with other ARV drug classes [20, 42–44]. In addition, DTG appears not to readily select for resistance mutations. DTG is now widely used in therapies for the treatment of both naïve and experienced patients [11, 12, 16, 18, 19]. However, patients in advanced clinical trials that were previously on a RAL-based therapy, who switched to a DTG-based salvage therapy, have shown signs of virological failure. In some cases, additional resistance mutations were selected [25, 26]. Therefore, there is a need for new INSTIs that can overcome emerging INSTI-resistant mutants.

BIC and CAB are now in late stage clinical trials [45–48]. Based on our antiviral analysis of the ability of these new INSTIs to inhibit previously identified INSTI-resistant single, double, and triple mutants in a single round replication assay, it appears that both BIC and CAB are both more broadly effective than either of the first generation INSTIs, RAL and EVG. However, in terms of their ability to inhibit the fifty-seven INSTI-resistant mutants we tested, BIC was significantly better than DTG against fourteen out of the mutants (Fig. 7; seven featured *p* values < 0.01, whereas four had *p* values < 0.001). BIC was also better than CAB against thirty-nine of the mutants (twenty-one had *p* values < 0.001). Conversely, DTG was better than BIC against ten of the mutants tested (four with *p* values < 0.001) and better than CAB for thirty-three of the mutants (fourteen with *p* values < 0.001). CAB was better than BIC and DTG for three mutants each (all three *p* values < 0.01 for DTG and two *p* values < 0.001 for BIC). Overall, our conclusions concerning the relative efficacies of the new INSTIs against mutants are in good agreement with the data of Yoshinaga et al. [41] and Neogi et al. [49], which appeared when this manuscript was in review.

Given the complexities of pharmacology, a significant difference in the behavior of a drug against a particular mutant (or mutants) may or may not translate directly into a desirable clinical outcome. However, given the problems that arise with drug resistance, it is likely that, among related compounds, those that are more broadly effective against resistant viruses will have an advantage in the clinic. In addition, in comparing the potencies of the compounds, the single round assay allows us to directly compare the efficacies of the new INSTIs against INSTI-resistant mutants in a reproducible and accurate manner. The single round assay avoids the issue of the effects of the mutations on the replication capacity, which, in turn, affects the number of viral life cycles in assays done with replication competent viruses, and by extension, can affect the EC_50_s.

Having a better understanding of how INSTIs bind to HIV-1 IN is an important part of developing more effective new drugs. However, superpositioning the available PFV and HIV IN structures has revealed differences in the PFV and HIV-1 IN active sites [15, 40]. Notably, the β4α2 loops are in different positions relative to the IN active site and there are differences in the structures and locations of the C-terminal domains (CTDs) near the IN active site. Thus, the contacts and interactions between INSTIs and the PFV intasome might not correspond exactly to the related contacts in the HIV-1 intasome. Until the structure of the HIV-1 intasome with these INSTIs bound is solved, HIV-1 IN models based on the structures of the PFV template with bound INSTIs and the available HIV-1 strand transfer (STC) structures can be used to predict how new INSTIs will bind to the HIV-1 intasome. DTG, BIC, and CAB are similar chemically and structurally. Not surprisingly, based on the model we built using the available structural information, all three compounds adopt similar configurations within the active site of HIV-1 IN. It appears that the structural differences on the “left side” of these INSTIs, the part of the pharmacophore away from the 3′ end of the viral DNA, are largely responsible for their different resistance profiles.

Although there are similarities, as noted above, BIC is better than DTG, and DTG is better than CAB, in terms of their respective abilities to broadly inhibit the known IN mutants. We think it is likely that BIC is more broadly effective in its ability to inhibit a range of INSTI-resistant mutants, because it is better able to adjust its conformation, in response to the changes in the shape of the active site caused by the various resistance mutations. Thus, as has been proposed for the binding of non-nucleoside reverse transcriptase inhibitors (NNRTIs) to HIV-1 RT [50–53], the most broadly potent compounds are those that are able to adjust their binding mode and/or their configuration in response to changes in and around the IN active site. As briefly discussed earlier, BIC has an oxazepine ring with a methylene bridge appended to its chelating scaffold, which differs from the oxazine ring of DTG. It appears, based on the models, that the oxazepine ring of BIC is more flexible, which would allow it to be more conformationally adaptable. The introduction of resistance mutations in residues in and around the IN active site may cause alterations in the active site geometry. These changes could potentially affect the binding of relatively rigid compounds, giving rise to resistance. The greater flexibility of the extended ring system of BIC may help it adapt to changes in the geometry in the IN active site, allowing BIC to overcome many of the known IN resistance mutations. However, the details of the binding of BIC, particularly to the INSTI-resistant forms of HIV-1 IN, will require additional high resolution structural data. Conversely, the methyl-modified oxazole ring of CAB does not appear to be in a favorable position to interact with WT IN. In addition, it does not appear to be conformationally adaptable. As a consequence, CAB may have difficulty overcoming the changes in the geometry of the active site of HIV IN caused by resistance mutants.

Generally speaking, the second generation INSTIs (DTG, BIC, and CAB) are much more proficient at inhibiting these INSTI-resistant mutants than RAL and EVG. Based on the data from our panel of mutants, DTG and BIC are more broadly effective against the mutants than CAB (Fig. 3). The potency of the second generation INSTIs can be affected by triple mutants which arise when mutations at G140 (A/C/S) and Q148 (H/R) are combined with the polymorphic mutation at L74M or T97A. Although it is not entirely clear how frequently L74M and T97A occur in either B or non-B HIV-1 subtypes in INSTI-naïve patients, it is possible that, when these polymorphisms are present, that they could affect the development of resistance.

## Conclusions

Based on these results, BIC appears to be a very promising INSTI. CAB has obvious disadvantages in terms of its breadth of antiviral potency relative to both BIC and DTG. However, CAB may have other advantages; it can be formulated as a long-acting compound that can be injected into patients once every 2–3 months [46, 47]. Nonetheless, based on experience with previous ARV drugs, in the long-term, resistant viruses will emerge. Thus, it is likely that at least some of the compounds that are broadly effective against the known mutants will be successful. This idea is underscored by the fact that, currently, in Washington DC, where the levels of HIV infection is still high, approximately 20% of new cases involve a HIV strain that has at least one drug resistance mutation [54].

### Methods

#### INSTI synthesis

Acquisition of RAL, EVG, and DTG was previously described [15, 55, 56]. BIC was obtained from PharmaBlock (Cat. No. PBLJ8958) and CAB was obtained from AbovChem LLC (Cat. No. HY-15592).

#### Cell-based assays

Single-round viral infectivity assays, using HIV-1 vectors that express either WT or mutant forms of IN, were used to determine antiviral potencies (EC_50_ values) of the compounds as previously described [57]. The Student’s *t* test was used to calculate the *p* values used to determine statistical significance.

#### Vector constructs

The vector pNLNgoMIVR-ΔENV.LUC has been described previously [36]. To produce the new IN mutants used in this study, the IN open reading frame was removed from pNLNgoMIVR-ΔENV.LUC by digestion with KpnI and SalI and resulting fragment was inserted between the KpnI and SalI sites of pBluescript KS+. Using that construct as the wild-type template, we prepared the following HIV-1 IN mutants using the QuikChange II XL site directed mutagenesis kit (Agilent Technologies, Santa Clara, CA) protocol: M50I, L74M, T97A, S119R, E138K, G140S, Q146L, Q146P, Q148H, Q148K, Q148R, S153Y, T66I/E157Q, E92Q/N155H, T124A/153Y, E138A/Q148R, E138K/Q148K, E138K/Q148R, E138K/263K, G140A/Q148H, G140A/Q148K, G140A/Q148R, G140C/Q148R, G140S/Q148K, G140S/Q148R, G140S/N155H, Y143H/N155H, Y143R/Q148H, Y143R/N155H, Q148H/N155H, Q148R/N155H, N155H/G163R, T66I/T97A/E157Q, L74M/G140A/Q148R, L74M/G140C/Q148R, E92Q/N155H/G163R, T97A/G140S/Q148H, T97A/Y143R/Q148H, T97A/Y143R/N155H, T97A/Q148H/N155H, E138A/G140S/Q148H, E138A/S147G/Q148R, E138K/G140A/Q148K, E138K/G140C/Q148R, E138K/G140S/Q148H, G140S/Y143R/Q148H, G140S/Y143R/N155H, G140S/Q148H/N155H, and G140S/Q148H/G163K. The following sense oligonucleotides were used with matching cognate antisense oligonucleotides (not shown) (Integrated DNA Technologies, Coralville, IA) in the mutagenesis: M50I, 5′-CAGCTAAAAGGGGAAGCCATTCATGGACAAGTAGACTGT-3′; T66I, 5′- ATATGGCAGCTAGATTGTATTCATTTAGAAGGAAAAGTT-3′; L74M, 5′- TTAGAAGGAAAAGTTATCATGGTAGCAGTTCATGTAGCC-3′; E92Q, 5′- GCAGAAGTAATTCCAGCACAAACAGGGCAAGAAACAGCA-3′; T97A, 5′- GCAGAGACAGGGCAAGAAGCTGCATACTTCCTCTTAAAA-3′; S119R, 5′- GTACATACAGACAATGGCCGTAATTTCACCAGTACTACA-3′; E138A, 5′- TGGGCGGGGATCAAGCAGGCTTTTGGCATTCCCTACAAT-3′; E138K, 5′- TGGGCGGGGATCAAGCAGAAATTTGGCATTCCCTACAAT-3′; G140A, 5′- GGGATCAAGCAGGAATTTGCTATTCCCTACAATCCCCAA-3′; G140C, 5′- GGGATCAAGCAGGAATTTTGTATTCCCTACAATCCCCAA-3′; G140S, 5′-GGGATCAAGCAGGAATTTTCCATTCCCTACAATCCCCAA-3′; Y143H, 5′- CAGGAATTTGGCATTCCCCATAATCCCCAAAGTCAAGGA-3′; Y143R, 5′-CAGGAATTTGGCATTCCCAGAAATCCCCAAAGTCAAGGA-3′; Q146L, 5′- GGCATTCCCTACAATCCCTTAAGTCAAGGAGTAATAGAA-3′; Q148H, 5′-TACAATCCCCAAAGTCACGGAGTAATAGAATCT-3′; Q148K, 5′- CCCTACAATCCCCAAAGTAAAGGAGTAATAGAATCTATG-3′; Q148R, 5′- CCCTACAATCCCCAAAGTCGTGGAGTAATAGAATCTATG-3′; S153Y, 5′- AGTCAAGGAGTAATAGAATATATGAATAAAGAATTAAAG-3′; N155H, 5′-GGAGTAATAGAATCTATGCATAAAGAATTAAAGAAAATT-3′; 5′-E157Q, 5′-ATAGAATCTATGAATAAACAATTAAAGAAAATTATAGGA-3′; G163K, 5′- GAATTAAAGAAAATTATAAAACAGGTAAGAGATCAGGCT -3′, G163R, 5′ -GAATTAAAGAAAATTATACGTCAGGTAAGAGATCAGGCT -3′, E138K for G140S/Q148H, 5′- TGGTGGGCGGGGATCAAGCAGAAATTTTCCATTCCCTACAATCCC-3′; S147G for E138A/Q148R, 5′- ATTCCCTACAATCCCCAAGGTCGTGGAGTAATAGAATCT-3′; E138K for G140C/Q148R, 5′- TGGGCGGGGATCAAGCAGAAATTTTGTATTCCCTACAAT-3′; E138K for G140A/Q148K, 5′- TGGGCGGGGATCAAGCAGAAATTTGCTATTCCCTACAAT-3′; E138A for G140S/Q148H, 5′- TGGGCGGGGATCAAGCAGGCATTTTCCATTCCCTACAAT-3′; Y143R for Y143R/Q148H, 5′-CAGGAATTTGGCATTCCCAGAAATCCCCAAAGTCACGGA-3′; Y143R for G140S/Q148H, 5′-CAGGAATTTTCCATTCCCAGAAATCCCCAAAGTCACGGA-3′; Y143R for G140S/N155H, 5′-CAGGAATTTTCCATTCCCAGAAATCCCCAAAGTCAAGGA-3′.

The following IN mutants from Fig. 2 (Additional file 1: Tables S1A and S1B), H51Y, T66I, E92Q, G118R, Y143R, N155H, R263K, H51Y/R263K, and G140S/Q148H have been described [35]. The remaining E138K/R263K double mutant was made using the previously constructed E138K mutant and the appropriate listed R263K oligonucleotides, which were used to add the second mutation.

The IN mutants from Fig. 4 (Additional file 1: Tables S2A and S2B), which includes M50I, L74M, T97A, S119R, E138K, G140S, Q146L, Q146P, Q148H, Q148K, Q148R, and S153Y, were constructed as described above using the appropriate listed oligonucleotides.

The IN mutants from Fig. 5 (Additional file 1: Tables S3A and S3B), were made as followed. The E138A/Q148R and E138K/Q148R double mutants were made using the previously generated Q148R mutant and the E138A and E138K oligonucleotides, respectively, to add the second mutation. The E138K/Q148K double mutant was constructed using the previously made E138K mutant and the appropriate Q148K oligonucleotides, which were used to add the second mutation. The G140A/Q148H and G140A/Q148K double mutants were made with the previously constructed G140A mutant and the appropriate oligonucleotides for the second mutation either Q148H or Q148K, respectively. The double mutants G140A/Q148R and G140C/Q148R were made with the previously generated Q148R mutant and the oligonucleotides for the second mutation, either G140A or G140C, respectively. The double mutants G140S/Q148K and G140S/Q148R were made using the previously generated G140S mutant and appropriate oligonucleotides for the second mutation, either Q148K or Q148R, respectively. The double mutants Q148H/N155H and Q148R/N155H were made using the previously generated N155H mutant and appropriate oligonucleotides for the second mutation, either Q148H or Q148R, respectively. The double mutant Y143R/Q148H was made using the previously generated Q148H mutant and appropriate oligonucleotides to introduce the second mutationY143R.

The IN mutants from Fig. 6 (Additional file 1: Tables S4A and S4B), were made as followed. The T66I/E157Q double mutant was made using the previously generated T66I mutant and the appropriate E157Q oligonucleotides to add the second mutation. The double mutants E92Q/N155H, G140S/N155H, Y143H/N155H, Y143R/N155H, and N155H/G163R were made using the previously generated N155H mutant and appropriate oligonucleotides for the second mutation, either E92Q, G140S, Y143H, Y143R, or G163R, respectively.

The IN mutants from Fig. 8 (Additional file 1: Tables S5A and S5B), were constructed as followed. The L74M/G140A/Q148R triple mutant was made using the previously generated G140A/Q148R double mutant and the oligonucleotides for the third mutation, L74M. The triple mutant L74M/G140C/Q148R was made with the previously generated G140C/Q148R double mutant and the oligonucleotides for the third mutation, L74M. The triple mutant T97A/Y143R/Q148H was constructed using the previously generated Y143R/Q148H double mutant and the appropriate oligonucleotides for the third mutation, T97A. The triple mutant E138K/G140C/Q148R was made using the previously generated G140C/Q148R double mutant and the appropriate oligonucleotides to create the third mutation, E138K. The triple mutant T97A/Q148H/N155H was made using the previously constructed Q148H/N155H double mutant and the appropriate oligonucleotides for the third mutation, T97A. The triple mutant E138A/S147G/Q148R was made with the previously generated E138A/Q148R double mutant and oligonucleotides to make the third mutation, S147G. The triple mutant E138K/G140A/Q148K was made using the previously constructed double mutant G140A/Q148K double mutant and the appropriate oligonucleotides to make the third mutation, E138K.

The IN mutants from Fig. 9 (Additional file 1: Tables S6A and S6B) were made as followed. The T66I/T97A/E157Q triple mutant was made using the previously generated T66I/E157Q double mutant and the oligonucleotides for the third mutation, T97A. The E92Q/N155H/G163R triple mutant was made using the previously generated E92Q/N155H double mutant and the oligonucleotides for the third mutation, G163R. The triple mutant G140S/Y143R/N155H was made using the previously constructed G140S/N155H double mutant and the correct oligonucleotides to create the third mutation, Y143R. The triple mutant T97A/Y143R/N155H was made with the previously generated Y143R/N155H double mutant and the appropriate oligonucleotides for the third mutation, T97A.

The IN mutants from Fig. 10 (Additional file 1: Tables S7A and S7B), were constructed as followed. The triple mutants T97A/G140S/Q148H, G140S/Q148H/N155H, and G140S/Q148H/G163K were each made with the previously generated G140S/Q148H double mutant and the appropriate oligonucleotides for the third mutation, either T97A, N155H, or G163K, respectively. The triple mutant was E138A/G140S/Q148H was made using the previously constructed G140S/Q148H double mutant and oligonucleotides to make the third mutation E138A. The triple mutant E138K/G140S/Q148H was made using the previously generated G140S/Q148H double mutant and the correct oligonucleotides to make the third mutation, E138K. The triple mutant G140S/Y143R/Q148H was made using the previously constructed G140S/Q148H double mutant and the appropriate oligonucleotides to make the third mutation Y143R.

The DNA sequence of each construct was verified independently by DNA sequence determination. The mutated IN coding sequences from pBluescript KS+ were then subcloned into pNLNgoMIVR-ΔEnv.LUC (between the KpnI and SalI sites) to produce mutant HIV-1 constructs; the sequence of the final construct was checked by DNA sequencing.

#### Computer modeling

All modeling was conducted using MOE 2016.0802 (Chemical computing group, Montreal, Quebec, Canada). The sequences and structures of DTG bound in the PFV intasome (PDB ID: 3S3M) and HIV-1 IN (PDB ID: 5U1C) served as the structural templates to construct a HIV-1 IN model with DTG bound in the active site. First, the N-terminal portions of the NTD, CCD, and CTD domains of the PFV and HIV-1 IN were used to align the domains properly. Next the sequences and structures of HIV-1 and PFV INs were aligned so that the HIV IN sequence was matched to superpose the HIV-1 and PFV IN. The coordinates of the HIV-1 IN structure (PDB ID: 5U1C) from the aforementioned alignment were used as the IN template to construct the HIV-1 IN model. This structure was modified to fit the structural coordinates of DTG, Mg^2+^ cofactors, and the viral DNA from the PFV intasome (PDB ID: 3S3M). The model of the HIV-1 intasome with DTG bound was energy minimized using a PFROSST forcefield with relative field solvation as recommended by the manufacturer. The new HIV-1 IN model was then aligned (structure only) with the HIV-1 IN structure (PDB ID: 5U1C) from the aforementioned alignment with PFV IN (PDB ID: 3S3M) and aligned to a RMSD value of 0.82 Å. Additionally, the new HIV-1 IN model was aligned with the previously solved HIV-1 IN structure (PDB ID: 5U1C) and aligned to a RMSD value of 1.12 Å. The surface (Van der Waals) of DTG was determined to locate possible steric clashes with the active site residues in the model. To identify potential contacts with CAB and BIC, both INSTIs were docked using DTG as the template. CAB or BIC were placed using the triangle matcher method and scored with London dG with approximately 30 poses and then the putative ligand poses were further refined using the rigid receptor method in MOE and scored with the GBVI/WSA dG function. If the expected ligand poses were not created, a pharmacophore editor tool in the docking function was used to add certain features that made the appropriate docking of CAB or BIC to DTG easier to view and the resulting structures were refined in the manner described above. The poses with the best docking scores were selected based on how well the bound compounds overlay with the DTG scaffold, bound to Mg^2+^, and how well their halogenated benzyl moiety interacted hydrophobically through π–π stacking with the penultimate cytosine on the 3′ end of the bound viral DNA. Docking poses images were refined using MolSoft ICM Pro software version 3.8-5 (MolSoft LLC, San Diego, CA).

## Additional file

**Additional file 1.** Two sets of supplementary tables are included for Figs. 2, 4, 5, 6, 8, 9, and 10. One set of tables (**A**) shows the antiviral activities of the INSTIs against INSTI-resistant mutants and the other set of tables (**B**) shows the statistical significance (*p* values) when comparing antiviral activities against INSTI-resistant mutants among the INSTIs.

## Abbreviations

INSTI’s: integrase strand transfer inhibitors
ARV: antiretroviral
STC: strand transfer complex
HIV: human immunodeficiency virus
PFV: prototype foamy virus
NNRTI’s: non-nucleoside reverse transcriptase inhibitors
FDA: Food and Drug Administration
EC_50_: half maximal inhibitory concentration
