# Supplementary material for: Efficacies of Cabotegravir and Bictegravir against drug-resistant HIV-1 integrase mutants
Source: Retrovirology. 2018 May 16;15:37. doi: 10.1186/s12977-018-0420-7 (PMC5956922; doi:10.1186/s12977-018-0420-7)
Supplement: Supplementary file 1 — Additional file 1. Two sets of supplementary tables are included for Figs. 2, 4, 5, 6, 8, 9, and 10. One set of tables (A) shows the antiviral activities of the INSTIs against INSTI-resistant mutants and the other set of tables (B) shows the statistical significance (p values) when comparing antiviral activities against INSTI-resistant mutants among the INSTIs. [file 12977_2018_420_MOESM1_ESM.pdf]

Supplemental Table 1

A

	WT	Y143R (RAL)	N155H (RAL)	G140S/Q148H (RAL)	T66I (EVG)	E92Q (EVG)	H51Y (DTG)	G118R (DTG)	R263K (DTG)	H51Y/R263K (DTG)	E138K/R263K (DTG)
**RAL**	4.0 ± 2.0	162.4 ± 16.2	153.6 ± 32.8	1900.0 ± 300.0	2.8 ± 0.4	29.8 ± 10.2	3.4 ± 0.2	35.5 ± 5.0	5.7 ± 2.3	6.0 ± 2.3	8.0 ± 1.6
**EVG**	6.4 ± 0.8	7.9 ± 2.3	90.0 ± 17.8	5700.0 ± 1100.0	66.2 ± 0.7	153.7 ± 34.0	4.5 ± 2.1	21.0 ± 9.5	5.4 ± 0.1	52.6 ± 18.2	11.4 ± 0.2
**DTG**	1.6 ± 0.9	4.3 ± 1.2	3.6 ± 1.3	5.8 ± 0.5	0.9 ± 0.8	2.3 ± 0.4	3.2 ± 0.2	13.0 ± 5.0	11.3 ± 3.4	16.0 ± 1.9	3.2 ± 0.5
**CAB**	2.4 ± 0.2	2.7 ± 0.6	2.2 ± 0.3	36.3 ± 6.5	0.9 ± 0.3	4.2 ± 0.1	9.8 ± 1.8	12.1 ± 1.9	13.4 ± 1.3	10.4 ± 1.5	4.2 ± 0.8
**BIC**	1.9 ± 0.3	2.6 ± 0.1	2.7 ± 0.8	5.5 ± 0.7	0.2 ± 0.1	2.0 ± 0.1	2.2 ± 0.4	5.4 ± 0.6	4.1 ± 1.1	3.5 ± 1.0	3.5 ± 0.6

B

		***p*-Value**
**WT**	DTG – CAB	NS
	DTG – BIC	NS
	CAB – BIC	NS
**Y143R**	DTG – CAB	NS
	DTG – BIC	NS
	CAB – BIC	NS
**N155H**	DTG – CAB	NS
	DTG – BIC	NS
	CAB – BIC	NS
**G140S/Q148H**	DTG > CAB	0.002
	DTG – BIC	NS
	BIC > CAB	0.002
**T66I**	DTG – CAB	NS
	DTG – BIC	NS
	BIC > CAB	0.01
**E92Q**	DTG > CAB	0.002
	DTG – BIC	NS
	BIC > CAB	< 0.001
**H51Y**	DTG > CAB	0.005
	BIC > DTG	0.009
	BIC > CAB	0.003
**G118R**	DTG – CAB	NS
	BIC > DTG	NS
	BIC > CAB	0.004
**R263K**	DTG – CAB	NS
	BIC > DTG	0.02
	BIC > CAB	< 0.001
**H51Y/R263K**	CAB > DTG	0.004
	BIC > DTG	< 0.001
	BIC > CAB	< 0.001
**E138K/R263K**	DTG – CAB	NS
	DTG – BIC	NS
	CAB – BIC	NS

Supplementary Tables 1A/B-7A/B. A. The EC50 values, with the standard deviations following the plus-minus sign (nM), were determined for RAL, EVG, DTG, BIC, and CAB against the INSTI-resistant mutants using single round infection assays (n=4). B. P values indicating statistically significant differences in the antiviral activities of the INSTIs for the various INSTI-resistant mutants. Dashes between the two INSTIs represent no significance differences in the data, whereas the ”>” sign indicates a significant difference in the antiviral activity of one INSTI over the other.

Supplemental Table 2

A

	M50I	L74M	T97A	S119R	E138K	G140S	Q146L	Q146P	Q148H	Q148K	Q148R	S153Y
**RAL**	30.7 ± 6.9	7.4 ± 2.1	2.9 ± 0.2	25.9 ± 0.1	6.5 ± 0.9	10.0 ± 3.7	24.1 ± 2.3	6.0 ± 0.8	142.2 ± 4.1	1450.0 ± 242.5	272.7 ± 7.0	18.0 ± 2.4
**EVG**	2.8 ± 0.4	6.0 ± 0.4	5.3 ± 0.4	7.6 ± 0.2	2.7 ± 0.4	12.6 ± 2.6	43.2 ± 5.4	4.7 ± 0.6	23.7 ± 4.2	309.8 ± 12.4	296.1 ± 24.2	4.2 ± 0.9
**DTG**	2.1 ± 0.9	2.2 ± 0.4	1.1 ± 0.5	2.3 ± 0.6	1.8 ± 0.4	2.7 ± 0.7	2.1 ± 0.8	0.5 ± 0.04	0.6 ± 0.1	1.0 ± 0.01	1.3 ± 0.2	2.0 ± 0.7
**CAB**	0.8 ± 0.1	0.9 ± 0.2	2.7 ± 0.7	2.3 ± 1.0	12.9 ± 1.0	5.1 ± 1.5	3.4 ± 0.6	10.3 ± 2.1	6.8 ± 1.5	2.9 ± 0.6	4.5 ± 1.3	3.2 ± 0.6
**BIC**	2.5 ± 0.3	1.4 ± 0.4	1.5 ± 0.3	3.3 ± 0.1	4.0 ± 0.2	3.5 ± 0.4	5.2 ± 1.3	0.8 ± 0.01	0.9 ± 0.1	1.2 ± 0.3	1.2 ± 0.3	2.8 ± 0.4

B

		***p*-Value**
**M50I**	DTG – CAB	NS
	DTG – BIC	NS
	CAB > BIC	< 0.001
**L74M**	DTG – CAB	0.003
	DTG – BIC	NS
	CAB – BIC	NS
**T97A**	DTG > CAB	0.01
	DTG – BIC	NS
	CAB – BIC	NS
**S119R**	DTG – CAB	NS
	DTG – BIC	NS
	CAB – BIC	NS
**E138K**	DTG > CAB	< 0.001
	DTG > BIC	< 0.001
	BIC > CAB	< 0.001
**G140S**	DTG – CAB	NS
	DTG – BIC	NS
	CAB – BIC	NS
**Q146L**	DTG – CAB	NS
	DTG > BIC	0.01
	CAB – BIC	NS
**Q146P**	DTG – CAB	0.003
	DTG – BIC	< 0.001
	CAB – BIC	0.003
**Q148H**	DTG > CAB	0.004
	DTG > BIC	0.01
	BIC – CAB	0.004
**Q148K**	DTG > CAB	0.02
	DTG – BIC	NS
	BIC > CAB	0.01
**Q148R**	DTG > CAB	0.02
	DTG – BIC	NS
	BIC > CAB	0.01
**S153Y**	DTG – CAB	NS
	DTG – BIC	NS
	CAB – BIC	NS

Supplemental Table 3

A

	G140A/ Q148H	Y143R/ Q148H	Q148H/ N155H	E138K/ Q148K	G140A/ Q148K	G140S/ Q148K	E138A/ Q148R	E138K/ Q148R	G140A/ Q148R	G140C/ Q148R	G140S/ Q148R	Q148R/ N155H
**RAL**	476.3 ± 22.0	2040.0 ± 99.0	337.5 ± 69.0	2885.0 ± 49.5	>5000	113.3 ± 1.4	3150.0 ± 210.0	3240.0 ± 876.8	>5000	>5000	>5000	>5000
**EVG**	250.8 ± 4.9	23.2 ± 5.7	620.9 ± 90.6	1890.0 ± 42.4	2053.3 ± 45.1	543.1 ± 89.2	293.2 ± 35.6	801.6 ± 67.7	575.0 ± 19.8	>5000	1326.3 ± 32.7	285.8 ± 18.2
**DTG**	3.9 ± 0.7	0.8 ± 0.2	4.0 ± 0.6	25.0 ± 2.1	450.7 ± 58.8	2.3 ± 0.2	4.4 ± 0.8	3.9 ± 0.1	4.1 ± 0.3	2.9 ± 0.6	26.2 ± 6.8	7.1 ± 2.0
**CAB**	3.9 ± 0.8	6.0 ± 0.4	13.3 ± 2.9	772.1 ± 72.2	393.1 ± 51.1	87.3 ± 7.6	25.6 ± 0.8	24.1 ± 0.1	13.7 ± 2.7	66.6 ± 8.1	414.6 ± 14.5	50.5 ± 6.5
**BIC**	1.1 ± 0.3	0.4 ± 0.1	4.0 ± 0.7	59.3 ± 4.9	137.1 ± 5.0	4.5 ± 0.4	4.1 ± 0.6	3.5 ± 0.4	10.0 ± 2.5	6.4 ± 1.4	6.1 ± 1.3	2.0 ± 0.4

B

		***p*-Value**
**G140A/Q148H**	DTG – CAB	NS
	BIC > DTG	0.002
	BIC > CAB	0.002
**Y143R/Q148H**	DTG > CAB	< 0.001
	BIC > DTG	0.02
	BIC > CAB	< 0.001
**Q148H/N155H**	DTG > CAB	0.006
	DTG – BIC	NS
	BIC > CAB	0.006
**E138K/Q148K**	DTG > CAB	< 0.001
	DTG > BIC	< 0.001
	BIC > CAB	< 0.001
**G140A/Q148K**	DTG – CAB	NS
	BIC > DTG	0.002
	BIC > CAB	0.002
**G140S/Q148K**	DTG > CAB	< 0.001
	DTG > BIC	< 0.001
	BIC > CAB	< 0.001
**E138A/Q148R**	DTG > CAB	< 0.001
	DTG – BIC	NS
	BIC > CAB	< 0.001
**E138K/Q148R**	DTG > CAB	< 0.001
	DTG – BIC	NS
	BIC > CAB	< 0.001
**G140A/Q148R**	DTG > CAB	0.005
	DTG > BIC	0.02
	CAB – BIC	NS
**G140C/Q148R**	DTG > CAB	< 0.001
	DTG > BIC	0.01
	BIC > CAB	< 0.001
**G140S/Q148R**	DTG > CAB	< 0.001
	BIC > DTG	0.008
	BIC > CAB	< 0.001
**Q148R/N155H**	DTG > CAB	< 0.001
	BIC > DTG	0.01
	BIC > CAB	< 0.001

Supplemental Table 4

A

	T66I/E157Q	E92Q/N155H	G140S/N155H	Y143H/N155H	Y143R/N155H	N155H/G163R
**RAL**	98.7 ± 20.2	>5000	477.8 ± 66.6	1205.0 ± 77.8	>5000	245.5 ± 43.8
**EVG**	67.5 ± 10.6	1601.0 ± 77.8	51.9 ± 10.8	70.0 ± 13.3	170.1 ± 34.0	72.6 ± 6.8
**DTG**	0.5 ± 0.1	1.8 ± 0.2	2.0 ± 1.0	1.7 ± 0.7	2.5 ± 0.8	1.6 ± 0.4
**CAB**	0.3 ± 0.1	4.6 ± 1.7	4.4 ± 0.6	1.2 ± 0.1	3.7 ± 0.2	2.2 ± 0.4
**BIC**	1.1 ± 0.2	3.3 ± 0.9	2.2 ± 0.5	2.7 ± 0.6	4.0 ± 0.8	2.1 ± 0.1

B

		***p*-Value**
**T66I/E157Q**	DTG – CAB	NS
	DTG > BIC	0.004
	CAB > BIC	0.001
**E92Q/N155H**	DTG – CAB	NS
	DTG – BIC	NS
	CAB – BIC	NS
**G140S/N155H**	DTG > CAB	0.01
	DTG – BIC	NS
	BIC > CAB	0.001
**Y143H/N155H**	DTG – CAB	NS
	DTG – BIC	NS
	CAB > BIC	0.01
**Y143R/N155H**	DTG – CAB	NS
	DTG – BIC	NS
	CAB – BIC	NS
**N155H/G163R**	DTG – CAB	NS
	DTG – BIC	NS
	CAB – BIC	NS

Supplemental Table 5

A

	T97A/Y143R/Q148H	T97A/Q148H/N155H	E138K/G140A/Q148K	L74M/G140A/Q148R	L74M/G140C/Q148R	E138K/G140C/Q148R	E138A/S147G/Q148R
**RAL**	>5000	4455.0 ± 388.9	>5000	>5000	>5000	>5000	1410.0 ± 183.8
**EVG**	41.6 ± 3.0	224.5 ± 24.8	4090.0 ± 641.0	747.4 ± 78.5	>5000	945.3 ± 176.4	774.9 ± 17.2
**DTG**	1.5 ± 0.1	2.4 ± 0.7	212.1 ± 46.0	12.0 ± 2.1	10.2 ± 1.3	5.3 ± 1.0	5.5 ± 1.3
**CAB**	5.3 ± 0.7	1.8 ± 0.6	610.3 ± 8.6	53.2 ± 14.8	220.3 ± 41.2	134.2 ± 0.3	4.0 ± 0.2
**BIC**	1.2 ± 0.7	1.8 ± 0.3	223.0 ± 30.7	11.7 ± 1.3	6.1 ± 0.9	8.2 ± 1.1	2.3 ± 0.0

B

		p-Value
**T97A/Y143R/Q148H**	DTG > CAB	0.001
	DTG – BIC	NS
	BIC > CAB	< 0.001
**T97A/Q148H/N155H**	DTG – CAB	NS
	DTG – BIC	NS
	CAB – BIC	NS
**E138K/G140A/Q148K**	DTG > CAB	< 0.001
	DTG – BIC	NS
	BIC > CAB	< 0.001
**L74M/G140A/Q148R**	DTG > CAB	0.01
	DTG – BIC	NS
	BIC > CAB	0.01
**L74M/G140C/Q148R**	DTG > CAB	0.002
	BIC > DTG	0.003
	BIC > CAB	0.002
**E138K/G140C/Q148R**	DTG > CAB	< 0.001
	DTG > BIC	0.008
	BIC > CAB	< 0.001
**E138A/S147G/Q148R**	DTG – CAB	NS
	BIC > DTG	0.02
	BIC > CAB	< 0.001

Supplemental Table 6

A

	T66I/T97A/E157Q	T97A/Y143R/N155H	G140S/Y143R/N155H	E92Q/N155H/G163R
**RAL**	33.5 ± 8.7	>5000	>5000	>5000
**EVG**	69.4 ± 11.8	642.7 ± 16.5	93.1 ± 16.1	677.9 ± 45.8
**DTG**	0.5 ± 0.1	8.5 ± 1.5	2.6 ± 0.3	3.8 ± 0.7
**CAB**	0.8 ± 0.1	142.2 ± 8.3	20.0 ± 3.5	4.2 ± 1.5
**BIC**	0.4 ± 0.2	8.2 ± 1.7	2.1 ± 0.1	2.0 ± 0.4

B

		***p*-Value**
**T66I/T97A/E157Q**	DTG > CAB	0.005
	DTG – BIC	NS
	BIC > CAB	0.02
**T97A/Y143R/N155H**	DTG > CAB	< 0.001
	DTG – BIC	NS
	BIC > CAB	< 0.001
**G140S/Y143R/N155H**	DTG > CAB	0.002
	DTG – BIC	NS
	BIC > CAB	0.002
**E92Q/N155H/G163R**	DTG – CAB	NS
	BIC > DTG	0.007
	CAB – BIC	NS

Supplemental Table 7

A

	T97A/G140S/Q148H	E138A/G140S/Q148H	E138K/G140S/Q148H	G140S/Y143R/Q148H	G140S/Q148H/N155H	G140S/Q148H/G163K
**RAL**	>5000	>5000	>5000	>5000	>5000	>5000
**EVG**	>5000	>5000	>5000	>5000	>5000	>5000
**DTG**	55.9 ± 3.0	13.8 ± 4.8	68.2 ± 2.0	7.7 ± 2.0	77.9 ± 15.9	24.3 ± 1.1
**CAB**	43.7 ± 4.2	70.2 ± 9.0	93.0 ± 6.1	113.8 ± 23.1	2423.3 ± 453.9	32.4 ± 3.1
**BIC**	29.5 ± 4.4	5.1 ± 0.5	4.9 ± 0.3	9.4 ± 0.3	57.5 ± 5.0	8.8 ± 1.9

B

		***p*-Value**
**T97A/G140S/Q148H**	CAB > DTG	0.004
	BIC > DTG	< 0.001
	BIC > CAB	0.003
**E138A/G140S/Q148H**	DTG > CAB	< 0.001
	DTG – BIC	NS
	BIC > CAB	< 0.001
**E138K/G140S/Q148H**	DTG > CAB	0.002
	BIC > DTG	< 0.001
	BIC > CAB	< 0.001
**G140S/Y143R/Q148H**	DTG > CAB	0.003
	DTG – BIC	NS
	BIC > CAB	0.003
**G140S/Q148H/N155H**	DTG > CAB	0.002
	DTG – BIC	NS
	BIC > CAB	0.002
**G140S/Q148H/G163K**	DTG > CAB	0.009
	BIC > DTG	< 0.001
	BIC > CAB	< 0.001
